# Regulation of protein arginine methyltransferase in osteoporosis: a narrative review

**DOI:** 10.3389/fcell.2025.1453624

**Published:** 2025-04-24

**Authors:** Ruiming Wen, Ruiqi Huang, Mianmian Yang, Jing Yang, Xuejie Yi

**Affiliations:** School of Sports Health, Shenyang Sport University, Shenyang, Liaoning, China

**Keywords:** protein arginine transferase, osteoporosis, bone formation, bone resorption, osteoclast, osteoblast

## Abstract

Osteoporosis (OP), a systemic bone disease characterised by increased bone fragility and susceptibility to fracture, is mainly caused by a decline in bone mineral density (BMD) and quality caused by an imbalance between bone formation and resorption. Protein arginine methyltransferases (PRMTs) are epigenetic factors and post-translational modification (PTM) enzymes participating in various biological processes, including mRNA splicing, DNA damage repair, transcriptional regulation, and cell signalling. They act by catalysing the transfer and modification of arginine residues and, thus, have become therapeutic targets for OP. In-depth studies have found that these enzymes also play key roles in bone matrix protein metabolism, skeletal cell proliferation and differentiation, and signal pathway regulation to regulate bone formation, bone resorption balance, or both and jointly maintain bone health and stability. However, the expression changes and mechanisms of action of multiple members of the PRMT family differ in OP. Therefore, this paper discusses the biological functions, mechanisms of action, and influencing factors of PRMTs in OP, which is expected to provide a new understanding of the pathogenesis of OP. Furthermore, we present theoretical support for the development of more precise and effective treatment strategies as well as for further study of the molecular mechanisms of PRMTs.

## 1 Introduction

Osteoporosis (OP) is a common bone disease that mainly manifests as bone loss, bone microstructure destruction, and increased bone fragility, which seriously affects the quality of life of patients (Foessl et al., 2023; Walker and Shane, 2023). The ageing population has been accompanied by an increased annual incidence of OP, which has become a global public health problem (Clynes et al., 2020). The pathogenesis of OP involves abnormal changes in bone homeostasis, which are affected by the complex mechanism of CO regulation in osteoblast (OB) differentiated from bone marrow mesenchymal stem cells (BMSCs). This process in addition to osteoclast (OC) generation after fusion of mononuclear macrophages, results in an imbalance between bone formation and resorption (Li et al., 2023a; Fischer and Haffner-Luntzer, 2022). The stimulation by various factors (such as ageing, oestrogen reduction, oxidative stress, and chronic inflammation) may trigger an imbalance in skeletal cell activity and aggravate OP development (Guo et al., 2021; Wu D. et al., 2021; Mkhize et al., 2023). Therefore, an in-depth study of the underlying pathogenesis of OP has important theoretical significance and clinical value for exploring effective prevention and treatment strategies.

With the rapid development of epigenetics, N6-methyladenosine (m6A) and protein methylation have gradually emerged (Yang et al., 2023; Wang et al., 2020). It has been reported that m6A, as the most abundant internal modification of mRNA, has been widely studied in OP. This involves the splicing, translation, and transcription of mRNA and affects the proliferation, differentiation, and function of bone cells by regulating the expression level of related genes (Feng et al., 2024; He et al., 2022; Wu et al., 2018). In recent years, Protein methylation has received increasing attention (Blanc and Richard, 2017). This dynamic methylation process is precisely regulated by histone methyltransferases and demethylases, which are involved in gene transcription (Hwang et al., 2021). Recently, attention has been focused on protein arginine methyltransferases (PRMTs), a nine-member class of crucial post-translational modification (PTM) enzymes found in mammals (Wu Q. et al., 2021; Zhu et al., 2024; Bedford and Clarke, 2009). These enzymes participate in mRNA splicing, RNA localisation, and PTM of proteins in the process of gene expression regulation (Hsu and Hung, 2023; Sachamitr et al., 2021; Bai et al., 2022). In addition, they also indirectly affect the complexity of cell signalling, fine regulation of the cell cycle, and the accurate response of DNA damage response (DDR) (Guccione and Richard, 2019). An increasing number of studies have indicated that abnormal PRMT expression is an independent risk factor for OP (Dashti et al., 2023). It is worth noting that an in-depth study of tissue-specific deletions has provided strong support for this view (Choi et al., 2021; Jang et al., 2021; Zhang et al., 2023). PRMTs precisely regulate the proliferation, differentiation, apoptosis, and other key biological processes of skeletal cells and affect the methylation reaction at the molecular level. Thus, PRMTs regulate the signalling of bone matrix proteins related to bone metabolism (Dashti et al., 2023; Dong et al., 2017; Vuong et al., 2024), and this subtle regulatory network plays a pivotal role in OP pathogenesis (Table 1). In this study, we analysed the expression changes and mechanisms of action of PRMTs in OP to highlight the key nodes in this complex network. Furthermore, we provide new potential ideas and methods for the prevention and treatment of OP.

**TABLE 1:** Phenotypic changes of protein arginine methyltransferases (PRMTs) affecting bone and related skeletal cells in osteoporosis (OP).

PRMTs	Research object	Phenotypic changes	Mechanism	The direction of effect	References
PRMT1	Prmt1^fl/fl^ mice, MC3T3-E1	Craniofacial malformation, cleft palate, craniofacial bone volume↓	Promote bone formation	Inhibiting OP	Gou et al. (2018a)
	MC3T3-E1, C57BL/6 mice	Skull atrophy, reduced mineral density, and incomplete suturing, Runx2, Alp, OCN↑	Promote the proliferation and differentiation of osteoblasts	Inhibiting OP	Ye et al. (2023)
	BMDM, OVX mice	c-Fos, NFATc1, TRAP, F- Actin ring formation↓, Bone microstructure and BMD↑	Inhibit osteoclast activity and bone resorption	Inhibiting OP	Choi et al. (2021); Choi et al. (2018)
	MC3T3-E1	Calcium deposition↓, Osteoblast markers change over time	Inhibition of osteogenic differentiation	Promoting OP	Dashti et al. (2023)
PRMT3	OVX mice, MSC	Runx2, OCN, Sp7, ALP activity and bone microstructure↑, Number of osteoclasts↓	Promote osteogenic differentiation of MSC cells	Inhibiting OP	Min et al. (2019)
PRMT4	CARM1-KO mice, BMSCs	ALP activity↑, Formation of mineralised nodules and calcium deposition↑	Promote osteogenic differentiation of BMSCs	Inhibiting OP	Li J. Y. et al. (2023)
	OVX mice, MC3T3-E1, BMDM, RAW264.7	OCN, Spp1, Col1a1, ALP activity↑, CTSK, c-Fos and Nfatc1↓, BMD and bone microstructure↑	Promotes osteogenic differentiation and impairs osteoclast differentiation	Inhibiting OP	Zhang et al. (2023)
	hBMSCs	Number of cell clones↑, β-Gal↓	Inhibition of cellular senescence	Inhibiting OP	Xu et al. (2019)
	MC3T3-E1	CCNB2, Number of cells in G2/M phase↑	Promote cell proliferation	Inhibiting OP	Dashti et al. (2023)
PRMT5	BMMs, Raw 264.7	CTSK, NFATc1, PU.1, F-actin ring formation and bone resorption↑	Promote osteoclast differentiation	Promoting OP	Ding et al. (2023)
	ST-2 and W-20	Runx2, Osx, OCN↓, CFU↑	Promote proliferation and inhibit osteogenic differentiation	Promoting OP	Kota et al. (2018)
	OVX mice, BMMs	Trap positive cells and actin ring formation↑, OPG in serum ↓, bone microstructure↓	Promote osteoclast differentiation	Promoting OP	Dong et al. (2017)
PRMT6	mice, MSC	RUNX2, Sp7, ALP↑, Calcium nodule↑	Promote osteogenic differentiation	Inhibiting OP	Li Z. et al. (2021)
PRMT7	C3H/10T1/2, BMSCs	Runx2, Col1a1, ALP, Sp7, OCN↑, Calcium nodule↑	Promote osteogenic differentiation	Inhibiting OP	Vuong et al. (2024)

↑: upregulation; ↓: downregulation.

## 2 Overview of PRMTs

Arginine methylation is a common protein PTM involved in regulating a variety of cellular processes (Li Y. et al., 2021; Lei et al., 2022). PRMTs catalyse the transfer of the methyl group of S-adenosylmethionine to the guanidino nitrogen atom of the arginine residue of the protein (Blanc and Richard, 2017; Hartley and Lu, 2020). This modification affects the structure, stability, and function of proteins and participates in regulating a wide range of cellular processes, such as gene expression, signalling, and protein degradation (Wei et al., 2014). According to the number and sites of modified methyl groups on arginine, arginine methylation modification is mainly divided into three types: ω-N^G^ monomethylarginine (MMA); ω-N^G^, N^G^ asymmetric dimethylarginine (ADMA); and ω- N^G^, N^G^-symmetric dimethylarginine (SDMA) (Lorton and Shechter, 2019). These derivatives constitute various proteins in the cytoplasm, nucleus, and organelles and together constitute a complex regulatory network inside the cell (Yin et al., 2023; Poulard et al., 2020; Repenning et al., 2021).

**FIGURE 1:**
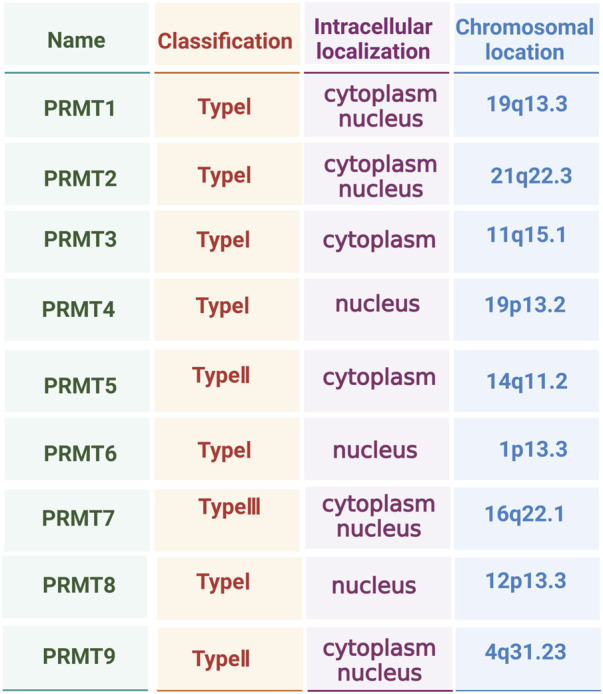
Type, intracellular localisation, and chromosomal location of protein arginine methyltransferase (PRMT) family members.

Presently, nine members of the PRMT family have been identified (PRMT1 to PRMT9), and PRMT proteins can be mainly divided into the following three categories according to the different catalytic methylation products. Type I PRMTs are responsible for the catalytic generation of MMA and ADMA and consist of PRMT1, PRMT2, PRMT3, PRMT4, PRMT6 and PRMT8. Type II PRMTs catalyse the generation of MMA and SDMA, and are PRMT5 and PRMT9. Finally, PRMT7 is classified as type III because it only catalyses the generation of MMA (Spadotto et al., 2020; Yang and Bedford, 2013) (Figure 1). Among these enzymes, type III exclusively produces the monomethylated arginine MMA, which is an intermediate product of ADMA and SDMA catalysed by type I and type II PRMTs (Lorton and Shechter, 2019). Protein sequencing of PRMT has revealed that its catalytic centre region has typical methyltransferase features, including I, post-I, II, and III motifs, which are common features of the seven-chain methyltransferase superfamily (Lorton and Shechter, 2019). In addition, the PRMT subfamily has two unique sequence motifs: double E (composed of two glutamate residues) and THW (composed of threonine–histidine–tryptophan) (Wang et al., 2022a; Debler et al., 2016). As epigenetic regulators of gene expression, PRMTs play important roles in both CO activation and the co-repression of transcription. PRMTs deposit key activating (H4R3me2a, H3R2me2s, H3R17me2a, and H3R26me2a) or repressing (H3R2me2a, H3R8me2a, H3R8me2s, and H4R3me2s) histone markers. It is noteworthy that the methylation status of arginine residues in downstream mediators of PRMTs directly determines the specific functions of PRMTs. In addition, methylation status also determines the amplitude and duration of signal transduction, and thereby accurately regulates cell proliferation, survival, differentiation and metabolic activities (Hwang et al., 2021; Andreu-Pérez et al., 2011; Hein et al., 2015). This regulatory mechanism is essential for maintaining normal physiological functions. The cellular localisation of PRMTs is also an important focus in PRMT research. Different PRMTs have different cell distributions and, therefore, modify different substrate proteins, producing varying forms of arginine methylation modifications.

Thus, PRMTs affect the function and activities of cells, and their diverse cellular localisation creates highly complex and flexible cellular regulatory networks (Price and Hevel, 2020; Morales et al., 2016; Hashimoto et al., 2020) (Figure 1). Although the importance of PRMTs and arginine methylation modifications in biology has long been recognised, relatively few studies have reported their specific functions and mechanisms of action, especially in OP. Consequently, we comprehensively explored existing literature on the regulatory network of PRMTs in the next section to provide a theoretical and experimental basis for developing new therapeutic methods and drugs.

## 3 PRMT1 and OP

PRMT1, the main arginine methyltransferase in mammals, accounts for approximately 85% of type I PRMTs (Tang et al., 2000). Human PRMT1 is encoded by the *PRMT1* gene located on chromosome 19 (19q13.3) and consists of 12 exons and 11 introns (Scorilas et al., 2000). This gene produces seven PRMT1 subtypes of proteins, and, therefore has obvious sequence specificity and substrate diversity (Thiebaut et al., 2021; Goulet et al., 2007). PRMT1 mainly catalysing the monomethylation (me1) and asymmetric dimethylation (me2a) of arginine side chains in proteins to participate in cellular processes such as transcriptional regulation, signal transduction, and DNA damage repair (Thiebaut et al., 2021; Yao B. et al., 2021).

### 3.1 PRMT1 and bone formation

New evidence has shown that PRMT1 is highly expressed in non-mesenchymal cell (MSC)-derived populations in calli, a variety of human musculoskeletal tissues (bone, cartilage, muscle, growth plate, and adipose tissue). It is also widely expressed in human BMSCs (hBMSCs) and mouse skull, frontal bone, and parietal bone (Dashti et al., 2023). Cranial neural crest (CNC)-specific PRMT1 deletion reduces the size, surface area, and volume of the craniofacial bone in mice, including the anterior maxilla, maxilla, palatal bone, frontal bone, mandible, and incisor and alveolar bone (Gou et al., 2018a).

In addition, the use of the small molecule inhibitor MS023 (PRMT1 inhibitor) leads to the reduction of the craniofacial complex length, expansion of suture space, cranial atrophy, reduction of mineral density, and incomplete suture (Ye et al., 2023). These observations indicate that PRMT1 plays an important role in bone growth. *In vitro* experiments have shown that silencing PRMT1 using MS023 treatment led to time-dependent morphological changes in MC3T3-E1 cells, which exhibited decreased activity, proliferation rate and differentiation ability. This was evidenced by a decreased proportion of cells in the G1 and S phases, calcium deposition, mineralised nodules and phosphate deposits, and expression of osteogenesis-related genes (Ye et al., 2023). These downregulated genes included Runt-related transcription factor 2 (RUNX2), alkaline phosphatase (ALP), osteocalcin (OCN) and osterix1 (SP7) (Ye et al., 2023).Although PRMT1 plays an important role in bone growth, some observed phenomena may also be influenced by other members of the PRMT family, such as PRMT3 and PRMT4.

Dashti et al. found that PRMT1 inhibited the osteogenic differentiation of MC3T3 cells. On day 10 following silencing PRMT1, the expression of integrin binding sialoprotein (IBSP) and phosphoethanolamine/phosphocholine phosphatase 1 (PHOSPHO1) significantly increased. However, the effect on ALP, biomineralisation associated (ALPL) expression or bone gamma carbohydrate protein (BGLAP) did not appear statistically significant at any time point. Interestingly, IBSP expression decreased because of the loss of PRMT1 on day 3 (Dashti et al., 2023). IBSP is considered an early marker of osteogenic differentiation (Zhang et al., 2009), which indicates that PRMT1 plays different roles at various stages of bone formation.

Pathological changes in OP are complex and precise processes, usually involving interactions between various cells and molecules (Wang et al., 2022b). Among them, bone morphogenetic protein (BMP), an important class of growth factors and cytokines, belongs to the transforming growth factor beta (TGF-β) superfamily, which mediates signal transduction by binding to dual receptor systems of type I and type II transmembrane serine/threonine kinase receptors, bone morphogenetic protein receptor type 1 (BMPR1) and BMPR2. This effect then modulates the SMAD signalling pathway to affect gene expression and the biological behaviour of primary OBs (Wu et al., 2016; Zou et al., 2021).

Considerable evidence shows that PRMT1 initiates BMP-induced SMAD signalling and is considered necessary for the biological response induced by BMP and, therefore plays a role in *Drosophila* wing and taste development (Xu et al., 2013; Gou et al., 2018b). PRMT1 short-interfering RNA (siRNA) transfection significantly downregulated the BMP target gene sarcomere, msh homeobox 1 (MSX1) in cranial bone-derived MC3T3-E1 cells and the frontal bone and mandible. This finding suggests that PRMT1 is an up-stream regulator of MSX1 in craniofacial bone development and promotes skull development (Gou et al., 2018a).

Inhibition of PRMT1 also reduced the expression of phosphorylated SMAD1/5/9 (p-SMAD1/5/9) in MC3T3-E1 cells and the histone H4 arginine 3 asymmetric dimethylation (H4R3me2a) activity of the PRMT1 substrate. Furthermore, it inhibited the viability, proliferation, and osteogenic differentiation of MC3T3-E1 cells, suggesting that PRMT1 promotes bone development by enhancing the phosphorylation signal transduction of BMP/SMADs and promoting the deposition of H4R3me2a markers (Ye et al., 2023). GSK715, a class I PRMT inhibitor, did not prevent the formation of H4R3me2a markers in MC3T3 cells (Dashti et al., 2023), which may be attributed to the compensatory activity of other PRMT members, including PRMT3 and PRMT4. To overcome the limitations of MS023 and GSK715 and more accurately evaluate the role of PRMT1 in bone growth and OB differentiation, more specific PRMT1 inhibitors need to be developed in future research. Through chemical synthesis or structural optimization, inhibitors that specifically target PRMT1 without affecting other PRMT members have been developed. These inhibitors will enable a more precise evaluation of the function of PRMT1 in the skeletal system.

### 3.2 PRMT1 and bone resorption

PRMT1 also affects the pathogenesis and development of OP by regulating bone resorption. Differentiation and increase of bone marrow macrophages (BMDM) following induction with colony-stimulating factor (M-CSF) and nuclear factor kappa B receptor activating factor ligand (RANKL), significantly increased the nuclear concentration of PRMT1 and its by-product, ADMA. This was accompanied by the formation of F-actin rings (Choi et al., 2018). Silencing PRMT1 reduced the number of tartrate-resistant acid phosphatase (TRAP)-positive multinucleated cells and downregulated the expression of OC-related genes, including proto-oncoprotein Fos (FOS), activated T cell nuclear factor 1 (NFATC1), cathepsin K (CTSK), TRAP, and F-actin. Moreover, this effect attenuated RANKL-induced osteoclastogenesis and bone resorption, which was also confirmed through PRMT1 haploid-deficient cell experiments (Choi et al., 2018). Animal studies have shown that haploinsufficiency of PRMT1 reduced TRAP activity and increased bone mineral density (BMD) and microstructure in ovariectomised (OVX) mice, suggesting that PRMT1 promotes bone resorption (Wang et al., 2022b). Melatonin injection significantly inhibited OC activity, bone microstructure damage, PRMT1, and OC marker expression in the bones of OVX mice, which was independent of the melatonin receptors, MT1/MT2 (Choi et al., 2021). Similarly, oestrogen treatment also inhibits bone resorption (Choi et al., 2018). These studies show that PRMT1 participates in the occurrence and development of OP by regulating osteoclastogenesis and bone resorption.

Notably, mitogen-activated protein kinases (MAPKs) are activated at the early stage of RANKL-induced OC differentiation and are responsible for activating the expression of related transcription factors and genes. Consequently, these kinases reduce the activity and number of OCs and the rate of bone resorption, and thereby achieve the purpose of treating OP (Iantomasi et al., 2023; Huang et al., 2023). MAPKs are a group of evolutionarily conserved serine/threonine kinases that can be divided into four subfamilies: extracellular signal-regulated kinase (ERK), P38 MAPK, c-Jun amino terminal kinase (JNK), and ERK5. These subfamilies represent the four canonical MAPK pathways (Peti and Page, 2013) and are important transmitters of signals from the cell surface to the interior nuclei. Consequently, they regulate cell growth, differentiation, environmental stress adaptation, inflammatory responses, and other important cellular, physiological, and pathological processes (Mao et al., 2023; Li et al., 2023b).

In the presence of M-CSF and RANKL, treatment of BMDMs with various inhibitors showed that inhibition JNK with SP 600125, but not p38 or ERK using SB 203580 or PD 98059, respectively, significantly reduced the nuclear expression of PRMT1 (Choi et al., 2018). This observation suggests that RANKL induced the expression of PRMT1 in OC differentiation through JNK signalling. The cytoplasmic tail of RANK contains three TRAF6 binding sites (Kondegowda et al., 2023). Silencing TRAF6 inhibited the protein and mRNA expression of PRMT1 in RANKL-stimulated BMDMs (Choi et al., 2021).

Following recruitment, TRAF6 activates a series of important signalling pathways, including NFKB, MAPK, and reactive oxygen species (ROS) signalling. These pathways activate the differentiation, survival, and proliferation of OCs and have a decisive impact on enhancing bone resorption (Huang et al., 2023; Yao Z. et al., 2021). PRMT1 siRNA inhibits the increase in NFKB activity in RAW264.7 cells, and RANKL treatment induces the interaction between PRMT1 and the P65 subunit (Choi et al., 2018), which suggests that TRAF6 plays a key role in regulating PRMT1-induced OC differentiation by RANK.

The above studies showed that PRMT1 positively regulates OC differentiation, survival, and bone resorption by regulating RANKL/TRAF6-mediated activation of JNK, NFKB, and other key signalling pathways and is involved in OP pathogenesis. Inhibiting PRMT1 or blocking the upstream RANKL/TRAF6 signalling reduces OC activity and bone resorption, suggesting that PRMT1 is a potential therapeutic target. The key role of PRMT1 in the pathogenesis of OP suggests that new drugs specifically regulating its activity should be developed. In addition, the mechanism of interaction between PRMT1 and downstream signalling pathways should be further studied to identify precise targets for drug design.

## 4 PRMT3 and OP

PRMT3 is a major functional cytosolic type I arginine methyltransferase that is mainly located in the cytoplasm. In addition to an arginine methyltransferase domain, it also has a “zinc finger” domain, which is different from that of other arginine methyltransferases, suggesting that PRMT3 may have unique functions (Frankel and Clarke, 2000; Tang et al., 1998; Landry-Voyer et al., 2023). A recent study reported that the expression levels of PRMT3 and H4R3me2a were significantly reduced in the BMSCs of OVX mice (Min et al., 2019). During the osteogenic differentiation of human MSCs (hMSCs), the expression levels of PRMT3 and H4R3me2a were significantly upregulated, accompanied by increased expression of the osteogenic marker gene *RUNX2* (Min et al., 2019).

Knockdown of PRMT3 can significantly decrease ALP activity, mineralised nodule formation (calcium deposition), and the expression of osteogenesis-related genes (*RUNX2* and *OCN*) and H4R3me2a in osteogenically induced hMSCs (Min et al., 2019). This observation suggests that PRMT3 regulates histone methylation through its arginine methyltransferase activity, thereby promoting the differentiation of hMSCs into OB. Tail vein injection of PRMT3 short hairpin RNA (shRNA) or a specific inhibitor (SGC707) induces osteopenia in mice and expression of the osteogenic genes *Runx2*, *Ocn*, and *Sp7*, ALP activity. In addition, this treatment also decreases bone microstructure, which is accompanied by an increase in the number of OC (Min et al., 2019).

Chromatin immunoprecipitation sequencing (ChIP-seq) identified miR-3648 as the co-binding site of PRMT3 and H4R3me2a, whereas PRMT3 knockdown reduced H4R3me2a levels in the promoter region of miR-3648. Moreover, overexpression of mir-3648 rescued the impaired osteogenic function in PRMT3 knockdown cells (Min et al., 2019). This observation suggests that PRMT3 activates miR-3648 expression by enhancing H4R3me2a levels in the gene promoter region, and thereby promotes osteogenic differentiation of MSCs. However, the specific molecular mechanisms underlying these actions require further investigation. Further research should analyse the molecular mechanism of this process using gene editing technology (such as clustered regularly interspaced short palindromic repeats [CRISPR]- CRISPR-associated protein 9 [Cas9]) and high-throughput sequencing technology to provide a theoretical basis for precision therapy.

## 5 PRMT4 and OP

PRMT4 was initially identified as coactivator-associated arginine methyl transfer 1 (CARM1) by Chen et al. and was the first PRMT known to have transcriptional coactivator functions (Chen et al., 1999; Suresh et al., 2021). The coding gene of the human PRMT4 protein is located on chromosome 19p13.2 and consists of 608 amino acid residues (Jin et al., 2023). PRMT4 is unique because it can be alternatively spliced to form a variety of isoforms, which differ in subcellular localisation, stability, substrate methylation levels, and binding affinity to target proteins. This diversity enables PRMT4 to exert various enzymatic activities in different tissues, further highlighting the richness of its functions (Jin et al., 2023; Xie et al., 2024). It coordinates chromatin remodelling, transcriptional regulation, mRNA splicing, and stability, contributing to the functional diversity of PRMT4 (Wang et al., 2014; Shin et al., 2016). In addition to the established involvement in transcriptional control, PRMT4-mediated methylation also affects a series of biological processes, including the cell cycle, metabolism, redox homeostasis, and inflammation (Zhang et al., 2023; Chen et al., 2024; Wang T. et al., 2022). This supports its possible role as a driver gene in the occurrence and development of OP.

Clinical data show that PRMT4 is highly expressed in the human bone and cartilage (Dashti et al., 2023). The single nucleotide polymorphism (SNP) rs6722613 of PRMT4 in OBs in women is closely related to the BMD of the femoral neck (FN) and lumbar spine (LS) (Panach et al., 2020). In addition, the expression of PRMT4 in the bones of patients with OP is significantly downregulated (Zhang et al., 2023). According to the Gene Expression Omnibus (GEO) database (GSE156508, GSE176265), the expression of PRMT4 in primary differentiated OBs and OCs of patients with OP who have fractures was significantly downregulated (Panach et al., 2020; Kim et al., 2021). The differential expression of this gene implies a close relationship with OP.

PRMT4 heterozygous knockout mice show significant bone loss, which manifests as impaired bone microstructure and decreased synthesis and accumulation of collagen at the injury site. These knockout mice also exhibited decreased ALP activity, decreased expression of osteogenesis-related genes, and obvious inflammatory infiltration (Li J. Y. et al., 2023). PRMT4 deletion reduced the expression of various histone-methylated H3R2me2a-, H3R8me2-, H3R17me2a-, H3R26me2a-, H3R63me2a- and H3R128me-related proteins in mouse bone and BMSCs and inhibit the differentiation and mineralisation of BMSCs (Li J. Y. et al., 2023).

Intramedullary injection of PRMT4 attenuated bone loss, bone microstructure disorder, and the expression of OC-related genes (*CTSK*, *RANKL*, and *NFATC1*) in OVX mice and enhanced the expression of osteogenesis-related genes *OCN* and secreted phosphoprotein 1 (*SPP1*) (Zhang et al., 2023). This indicates that PRMT4 overexpression inhibited bone loss in OVX mice and similar results have been obtained *in vitro*. PRMT4 overexpression in MC3TE-E1 cells significantly increased the expression of the osteogenic-related genes *BGLAP*, *RUNX2*, and *ALPL* (Dashti et al., 2023), ALP activity, and extracellular matrix mineralisation in a time-dependent manner. PRMT4 overexpression in BMDM and RAW264.7 cells inhibits the expression of CTSK, c-Fos, and NFATc1 (Zhang et al., 2023), suggesting that it promotes osteogenic differentiation and inhibits OC differentiation.

Xu et al. (2013) found that overexpression of PRMT4 delayed cellular senescence in late-passage cells. The mechanism by which PRMT4 directly binds to the promoter region of the discoidin domain receptor 2 (DDR2) gene induces an increase in the methylation level of histone H3 arginine 17 residue (H3R17) at this site, thus upregulating the expression of DDR2 (Xu et al., 2019). Conversely, inhibition of PRMT4-mediated histone arginine methylation modification reduced the expression of DDR2 in early passage human BMSCs (hBMSCs), resulting in accelerated cell senescence (Xu et al., 2019).

Another study found that PRMT4 deletion reduced the expression of the mitosis-related marker cyclin B2 (CCNB2) and the proportion of cells in the G2/M phase in MC3T3 OB precursor cells (Dashti et al., 2023). This finding suggests that PRMT4 is involved in regulating cell proliferation. In summary, PRMT4, as a transcriptional coactivator, regulates the differentiation and function of OBs and OCs by coordinating chromatin remodelling, transcriptional regulation, and other mechanisms. In addition, it also participates in cell cycle regulation and cellular senescence, and plays an important role in maintaining bone homeostasis and in the pathogenesis of OP.

In addition, PRMT4 monitors cellular glucose metabolism and reprograms oxidative phosphorylation (OXPHOS) into the aerobic glycolytic pathway (Liu et al., 2017). In MC3T3-E1 and RAW264.7 cells, PRMT4 overexpression significantly increased the extracellular acidification rate (ECAR). In contrast, PRMT4 knockdown significantly increased the cell oxygen consumption rate (OCR) and mitochondrial membrane potential, whereas decreasing lactate content (Zhang et al., 2023) and these observations suggest that PRMT4 regulates glycolytic flux.

Metabolomic analysis revealed that fructose 1,6-diphosphate was the only upregulated metabolite in two PRMT4 overexpressing cell lines, and (E)-1-(pyridin-4-yl)-3-(quinolin-2-yl)prop-2-en-1-one (PFK15), the 6-phosphofructo-2-kinase/fructose-2,6-biphosphatase 3 (PFKFB3) inhibitor, reversed the effects of PRMT4 in promoting osteogenic differentiation (Zhang et al., 2023). This observation suggests that PRMT4 mediates OB/OC metabolic reprogramming by increasing the activities of phosphofructokinase 1 (PFK1) and PFKFB3) to regulate glycolytic flux.

Further studies have revealed that PFK1 and PFKFB3 were activated by serine/threonine kinase (AKT) and AMP-activated protein kinase (AMPK) phosphorylation. PRMT4 overexpression upregulates p-AKT (Thr450, Thr308, and Ser473) in mouse OBs and MC3T3-E1 cells and phosphorylates AMPK-Thr172 in RAW264.7 cells, resulting in increased phosphorylation of PFK1 and PFKFB3 (Zhang et al., 2023). However, MK2206, an AKT inhibitor, and compound C, an AMPK inhibitor, reversed the regulatory effect of PRMT4 on PFK activity. Co-immunoprecipitation experiments have confirmed that PRMT4 interacts with protein phosphatase 1 CA (PPP1CA) and increases with increasing protein concentrations (Zhang et al., 2023). This observation indicates that PRMT4 may affect AKT-T450 and AMPK-T172 dephosphorylation by methylating the PPP1CA R23 site, thereby upregulating glycolytic flux (Zhang et al., 2023).

## 6 PRMT5 and OP

PRMT5 is a major type II arginine methyltransferase that exists in eukaryotes (Stopa et al., 2015). It is mainly located in the cytoplasm (Rho et al., 2001) and catalyses the symmetric dimethylation of histone H2AR3, H3R8, H3R2, and H4R3 sites (Stopa et al., 2015). PRMT5 is a key protein expressed in MSCs that plays an important role in maintaining MSC homeostasis and promoting their differentiation into OBs. The PRMT5 inhibitor (GSK3235025) attenuated the proliferative ability of st-2 and W-20 cells and increased OB differentiation (Kota et al., 2018). Although this inhibitor does not change the total PRMT5 protein content, it prevents specific protein modification processes, such as global symmetric dimethylation of H3R8 and H4R3, without affecting H3R2 modification (Kota et al., 2018). Additionally, PRMT5 inhibition significantly reduced the intrinsic expression of interferon (IFN)-stimulated genes (ISG), and this effect was interestingly effectively blocked by externally introduced type I IFN signalling (Kota et al., 2018).

In RANKL-induced bone marrow mononuclear (BMM) cells, PRMT5 expression was upregulated in a time-dependent manner. Silencing PRMT5 or administering the PRMT5 inhibitor EPZ015666 (EPZ) inhibited multinucleated OCs and bone resorption, as well as the expression of the OC markers c-Fos, CTSK, NFATc1, TRAP, and matrix metalloproteinase 9 (MMP9) (Dong et al., 2017; Ding et al., 2023). Additionally, EPZ015866 has been reported to be superior to EPZ015666 in inhibiting the formation and resorption of mature OCs (Ding et al., 2023). Intraperitoneal injection of EPZ015666 increases bone mass and serum osteoprotegerin (OPG) levels in OVX mice and reduces the number of OC in bone and the levels of serum type I collagen cross-linked deaminase peptide (CTX-I) and RANKL (Dong et al., 2017).

These findings suggest that PRMT5 activates OC differentiation. Mechanistically, EPZ015666 treatment altered the expression of several OC formation-related genes, including chemokine C-X-C motif ligand 10 (*CXCL10*) and S-adenosyl methionine domain containing 2 (*RSAD2*). Administration of recombinant CXCL10 partially reversed the inhibitory effect of the PRMT5 inhibitor on osteoclastogenesis, and a similar knockdown of RSAD2 using siRNA inhibited RANKL-induced OC differentiation (Dong et al., 2017). Subsequent ChIP-qPCR revealed that both PRMT5 inhibition and knockdown reduced H3R8 or H4R3 methylation or both, of the CXCL10 and RSAD2 promoters (Dong et al., 2017).

These results illustrate that PRMT5 inhibition partially inhibits osteoclastogenesis by downregulating CXCL10 and RSAD2 expression. In addition, EPZ015666 also inhibited RANKL-induced NFKB and reduced the increased p-P38 and p-ERK levels but did not affect p-JNK levels (Dong et al., 2017). This effect inhibits the nuclear translocation of NFKB by inhibiting dimethylation of the P65 subunit, ultimately preventing OC differentiation and bone resorption (Ding et al., 2023). However, this has not yet been verified under pathological conditions.

In summary, PRMT5 is a type II arginine methyltransferase that plays a bidirectional regulatory role in maintaining the homeostasis of MSCs and regulating their differentiation into OBs and OCs by catalysing histone methylation. PRMT5 inhibitors can affect the expression of related genes, thereby inhibiting OC formation and bone resorption; however, they can also promote osteogenic differentiation.

## 7 PRMT6 and OP

PRMT6 is a type I PRMT mainly localised in the nucleus, with a molecular weight of approximately 41.9 kDa, and is located on human chromosome 1 (Frankel et al., 2002; Gupta et al., 2021). It can asymmetrically dimethylate arginine residues on protein substrates, which plays a key role in coordinating the epigenetic regulation of the expression of various genes (Chen et al., 2023). The expression of PRMT6 in the MSC-induced osteogenic lineage was significantly upregulated and positively correlated with the mRNA expression of RUNX2 and SP7. Knockdown of PRMT6 significantly reduced ALP activity, calcium nodules, and RUNX2 and SP7 expression (Li Z. et al., 2021).

Transcriptomics and RNA-seq revealed that PRMT6 was directly regulated by the RNA demethylase alkB homolog 5, RNA demethylase (ALKBH5) and was negatively correlated with its expression. Inhibition of ALKBH5 expression decreased the degradation rate of PRMT6 in an m6A-dependent manner and negatively regulated the osteogenic differentiation of MSCs, thereby reducing bone mass in mice (Li Z. et al., 2021). It is noteworthy that ALP activity and RUNX and SP7 expression decreased after the simultaneous knockdown of ALKBH5 and PRMT6. However, simultaneously overexpressing both ALKBH5 and PRMT6 effectively reversed these changes (Li Z. et al., 2021), indicating that PRMT6 is a key downstream target of ALKBH5 in inhibiting the osteogenic differentiation of MSCs.

In addition, the knockdown of ALKBH5 expression increased AKT signalling (Li Z. et al., 2021). Further studies showed that inhibition of the phosphatidylinositol-4,5-bisphosphate 3-kinase (PI3K)/AKT pathway using the inhibitor LY294002 significantly reduced gene expression related to osteogenesis. However, this effect was reversed by ALKBH5 overexpression in combination with activation of the PI3K/Akt pathway (SC79). Furthermore, silencing PRMT6 expression reduces p-AKT levels (Li Z. et al., 2021). These results suggest that the ALKBH5–PRMT6 axis regulates MSC osteogenesis mainly by activating the PI3K/AKT pathway. However, current research is still in its infancy, and numerous potential molecular mechanisms require further exploration and elucidation.

## 8 PRMT7 and OP

In 2008, Buhr et al. identified a novel arginine methyltransferase, PRMT7, through proteomics, which was shown to be a candidate protein related to pluripotency (Buhr et al., 2008). PRMT7 is responsible for maintaining pluripotency, proliferation, and differentiation of stem cells (Wang et al., 2021). Furthermore, it was considered the only type III protein arginine methyltransferase responsible for catalysing the formation of MMA, with unique substrate specificity (Morales et al., 2016; Miranda et al., 2004; Jain and Clarke, 2019). A clinical study in patients with intellectual disability syndrome found that those with PRMT7 mutations showed a short stature and brachydactyly (Valenzuela et al., 2019; Agolini et al., 2018), indicating that PRMT7 may be closely related to bone health.

*In vitro* experiments showed that PRMT7 deletion significantly reduced the expression of RUNX2, type I collagen alpha 1 chain (COL1A1), ALP, SP7, and OCN, as well as calcium deposition in C3H/10T1/2 cells and mouse-derived BMSCs (Vuong et al., 2024). This finding indicates that PRMT7 deletion weakened the differentiation ability of BMSCs. A delay in C3H/10T1/2 cell differentiation led to a continuing weakening of the pro-osteogenic function of PRMT7 (Vuong et al., 2024). This observation indicates a critical role for PRMT7 in the early stage of osteogenic differentiation, but it has different roles in late-stage osteogenesis.

Mechanistically, BMP4 application to PRMT7-depleted cells continuously increased the levels of p-SMAD1 and SMAD1/5/8 proteins and MMA content on the surface of bone morphogenetic protein receptor 1A (BMPR1A [ALK3]). In contrast, in the absence of BMP4 treatment, SGC-8158 (a PRMT7 inhibitor) decreased the MMA, p-SMAD1, and ALK3 levels, whereas attenuating the BMP4-induced nuclear localisation of p-SMAD1 (Vuong et al., 2024). This observation indicates that PRMT7 positively regulates the BMP signalling pathway by interacting with BMPRI, leading to arginine methylation of the receptor, which in turn affects the osteogenic differentiation of BMSCs.

## 9 Research limitations of PRMTs regulating OP

Although this paper systematically discusses the regulatory mechanisms of PRMTs in OP and their potential therapeutic targets, there are still some limitations and shortcomings that cannot be ignored. First, most existing studies are based on *in vitro* cell experiments and animal models. Although these models provide valuable insights into the biological functions of PRMTs, the results may not be fully extrapolated to humans because of the complexity of the internal environment, particularly the multi-system interactions in the human body. In addition, the limitations of the research scope should not be overlooked. The roles of other members of the PRMTs family, such as PRMT2, PRMT8, and PRMT9, in OP remain underexplored, and their potential regulatory mechanisms are not yet well understood. Current research mainly focuses on a few classical signalling pathways, such as BMP/Smad and RANKL/NF-κB pathways (Ye et al., 2023; Choi et al., 2018). However, studies on the correlation between PRMTs and key signalling networks, such as Wnt/β-catenin, Notch, and Hippo, are still insufficient, limiting a comprehensive understanding of the roles of PRMTs in OP. Regarding research methods and experimental approaches, the existing techniques also have limitations. Currently used PRMT inhibitors, such as MS023 and EPZ015666, have low target specificity, potentially inhibiting the activities of multiple PRMT family members. This makes it challenging to accurately evaluate the specific functions of individual PRMTs (Dashti et al., 2023; Ye et al., 2023). In the future, with the help of structural biology technology to analyze the active pockets of PRMTs, combined with computational simulation technology, it is expected to design highly selective inhibitors of PRMTs, thus improving the accuracy of targeted therapy. Secondly, using CRISPR/Cas9 gene editing technology to construct a conditional knockout mouse model, combined with high content screening method, we can systematically evaluate the function of a single PRMTs and deeply understand its specific mechanism of action in organisms. In addition, the specific binding patterns of PRMTs family members can be revealed through the study of single cell epigenetics, which provides a key basis for the design of targeted inhibitors and helps to develop more effective PRMTs inhibitors. However, the current research also has shortcomings in data interpretation. The existing chromatin immunoprecipitation sequencing (ChIP-seq) and transcriptome sequencing (RNA-seq) data mostly stay at the level of correlation analysis, lacking in-depth causal verification and functional experimental support. This makes the understanding of PRMTs regulatory network not deep enough, and it is difficult to fully reveal its complex regulatory mechanism in organisms. Therefore, future research needs to strengthen causal verification and functional experiments in order to better understand the regulatory network of PRMTs.

In terms of dynamic and spatiotemporal regulation of signalling pathways, the dynamic changes in PRMT expression across different tissues, such as bone marrow, bone cortex, and blood, during various stages of OP have not been systematically studied. Additionally, there is a lack of dynamic monitoring data with spatiotemporal resolution. Although the critical role of PRMTs as epigenetic regulators in bone formation and resorption has garnered significant attention (Dong et al., 2017; Li Z. et al., 2021), the interaction mechanisms between PRMTs and other epigenetic modifications, such as DNA methylation, histone acetylation, and m6A, remain unclear. m6A modification has been reported to exert broad functional effects in maintaining homeostasis, particularly through its involvement in the regulation of post-transcriptional gene expression, growth, and development (Zhao et al., 2017; Dorn et al., 2019). It not only participates in bone development but also plays a key role in the dynamic processes of “writing” and “erasing” modifications in OP (Huang and Wang, 2022). The m6A methyltransferase METTL3 and demethylase FTO are involved in the intricate balance between adipogenesis and osteogenesis, which is crucial for the pathological progression of OP (Wu et al., 2018; Chen et al., 2019; Li et al., 2019). Therefore, the mechanism by which m6A and PRMTs jointly regulate OP requires further investigation.

In clinical applications, although the therapeutic potential of PRMTs has been demonstrated in *in vitro* and animal models, their translation to clinical use faces significant challenges. At present, it is necessary to learn from the clinical trial data of PRMTs inhibitors in other diseases (such as cancer and neurodegenerative diseases) to evaluate their safety and the feasibility of transferring their efficacy to OP. For example, the study of GSK3326595 in AML reveals the dose-limiting toxicity of PRMT5 inhibitors (Watts et al., 2024), which may provide a reference for dose optimization in OP treatment. In addition, by integrating multiple omics data, it is helpful to identify the genetic variation of PRMT related to OP, which is helpful to develop individualized treatment schemes. In the future, it is necessary to explore the combined treatment strategy of PRMT inhibitors and existing anti-OP drugs, or to evaluate the activity of inhibitors (such as GSK3368715) that have entered clinical trials in bone cells, so as to promote their reuse in OP treatment. The extensive conservation and versatility of PRMT family members result in a wide range of non-targeted biological effects *in vivo*, which may lead to off-target effects and associated side effects of targeted therapies (Jarrold and Davies, 2019). Currently, most studies focus on the general regulatory mechanisms of PRMTs, with limited consideration for individualised treatment. The genetic polymorphisms of PRMTs in different patient populations and their potential impact on therapeutic outcomes remain unclear, posing a major obstacle to the advancement of precision medicine and personalised treatment.

## 10 Summary and outlook

**FIGURE 2:**
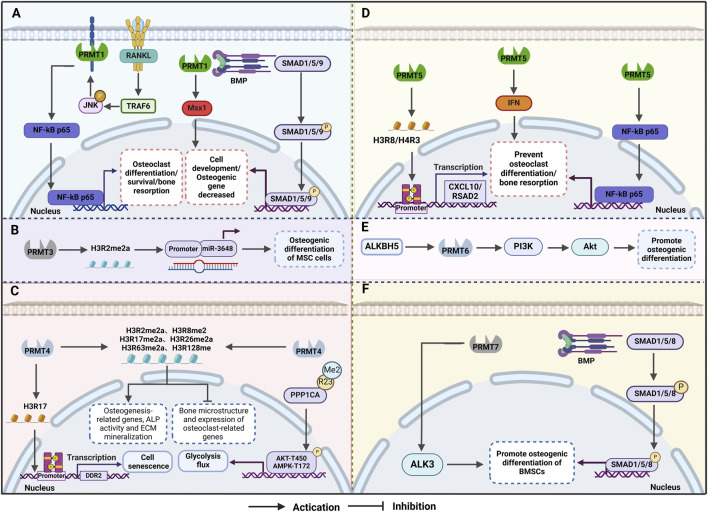
Mechanism of osteoporosis (OP) regulation by protein arginine methyltransferases (PRMTs) **(A)**PRMT1 influences bone formation and resorption through the BMP2/Smad and other pathways, thereby mitigating OP. **(B)** PRMT3 enhances the expression of miR-3648 by increasing H4R3me2a levels in the gene promoter region, promoting osteogenic differentiation of mesenchymal stem cells. **(C)** PRMT4 inhibits osteoclast differentiation and delays cellular ageing by enhancing glucose metabolism and glycolysis flux during osteogenic differentiation. **(D)** PRMT5 maintains homeostasis in mesenchymal stem cells and regulates their differentiation into osteoblasts and osteoclasts by catalysing histone methylation modifications. **(E)** ALKBH5 accelerates the degradation of PRMT6 mRNA in an m6A-dependent manner, with the ALKBH5-PRMT6 axis regulating MSC osteogenesis primarily through activation of the PI3K/Akt pathway. **(F)** PRMT7 positively regulates the BMP signalling pathway by interacting with BMPR type I receptors, leading to arginine methylation of the receptor and promoting osteogenic differentiation of bone marrow stromal cells.

In summary, PRMTs, a class of biomolecules that are important in epigenetic regulation and PTM, play a regulatory role in OP at multiple levels. The regulatory effects of these biomolecules involve catalysing the methylation of arginine residues and other transfer and modification processes. Studies have shown that a variety of PRMT enzymes show differential expression patterns in the bone tissues of patients with OP, and mainly include PRMT1, PRMT3, PRMT4, PRMT5, PRMT6, and PRMT7. These differential expression patterns imply that PRMTs play complex roles in the pathogenesis of OP. They affect a variety of biological processes in skeletal cells, including cell proliferation, differentiation, and energy metabolism, through the catalytic methylation of arginine residues. In the current study, we highlighted that only PRMT5 has a negative effect on this process (Figure 2) and, therefore, its dysregulation leads to impaired bone health.

Although the role of PRMTs in OP has been initially elucidated, many details and mechanisms remain to be further explored and studied. First, future research should expand the scope of studies on the PRMT family, particularly the potential regulatory mechanisms of PRMT2, PRMT8, and PRMT9, and their correlation with OP. The dynamic expression characteristics of PRMTs in different types of bone tissues and cell types should be thoroughly analysed using Qualcomm’s high-throughput screening tools and multi-omics integration approaches, such as proteomics, transcriptomics, and metabolomics. In addition, future studies should move beyond the single signalling pathway paradigm and construct interactive network models that integrate PRMTs with Wnt, Notch, and Hippo pathways to uncover their roles within broader regulatory signalling networks. With the rapid advancements in epigenetics, the interaction mechanisms between PRMTs and m6A in OP remain an underexplored area, presenting significant potential for future research. Given the differences in the expression patterns of PRMTs and their effects on OP, more attention should be directed toward individualised treatments. Precise, personalised treatment plans could be developed by assessing PRMT expression levels in patients and integrating this information with other biomarkers. Furthermore, future drug research and development will focus on PRMTs as therapeutic targets, with efforts directed toward designing small-molecule regulators to restore the balance between bone formation and bone absorption. Beyond traditional drug therapies, PRMTs may also serve as biomarkers for early diagnosis and prognosis evaluation in OP. Innovative approaches, such as cell and gene therapies based on PRMTs, are expected to enter clinical trials, offering patients a broader range of treatment options.
